# Clinicians' Experiences of Eating Disorder Focused Family Therapy With Autistic Young People

**DOI:** 10.1002/erv.3173

**Published:** 2025-01-20

**Authors:** Fiona Duffy, Imogen Peebles, Emma Clark, Rachel Loomes, Lisa Thomson, Ellen Maloney, Emy Nimbley

**Affiliations:** ^1^ Department of Clinical & Health Psychology University of Edinburgh Edinburgh UK; ^2^ NHS Lothian Child and Adolescent Mental Health Service Edinburgh UK; ^3^ Eating Disorders and Autism Collaborative (EDAC) University of Edinburgh Edinburgh UK; ^4^ South West London and St George's Mental Health NHS Trust London UK; ^5^ Department of Clinical Educational and Health Psychology University College London Research London UK

**Keywords:** anorexia nervosa, family based treatment, family therapy, out‐patient, treatment

## Abstract

**Objective:**

Eating disorder focused family therapy (FT‐ED) is the leading outpatient intervention for adolescents with Anorexia Nervosa. Autistic people report poorer eating disorder treatment experiences and may be at increased risk of inpatient admissions. There is a need to consider adaptions to eating disorder treatment for this population. The aim of this study is to explore the experiences of clinicians in the delivery of FT‐ED for Autistic young people with Anorexia Nervosa and any adaptations currently being implemented.

**Method:**

FT‐ED trained clinicians who had experience of delivering this modality with young Autistic people and their families, were invited to take part in interviews. Transcripts were analysed using Reflexive Thematic Analysis.

**Results:**

Eleven clinicians completed interviews and analysis generated four themes and eight subthemes: (1) Systemic context, (2) Raising potential autism, (3) Autism eating disorders crossover, (4) Manual versus adaptations.

**Conclusions:**

This paper is the first exploration of clinician's experience delivering FT‐ED to Autistic young people and their families and highlighted unique considerations with this population. It is an initial step to consider adaptations to the FT‐ED model, with the aim of making eating disorder treatments more effective, accessible and acceptable for Autistic young people and their families.

**Summary**

- FT‐ED clinicians highlighted a number of unique considerations when delivering this modality to Autistic young people, including the increased prevalence of neurodivergence within families and risk of missattunement within the systemic context.
- FT‐ED clinicians highlighted the late identification of autism in young people presenting to eating disorder services and the added complexity of differentiating between Autistic traits and an eating disorder.
- Finally, FT‐ED clinicians described tension on whether to adhere to a manualised approach or to make adaptions to treatment, underpinned by a fear of causing harm.

## 1 Introduction

Eating disorder focused family therapy (FT‐ED) is the leading outpatient intervention for adolescents with Anorexia Nervosa (AN) and is significantly superior to individual therapy on weight outcomes at the end of treatment (Austin et al. 2024). FT‐ED is internationally recommended by clinical treatment guidelines (Couturier et al. 2020; Crone et al. 2023; NICE 2017, SIGN 2022). Within the United Kingdom, these recommendations are implemented via either Family Based Treatment (FBT, Lock and Le Grange 2012) or AN focused family therapy (FT‐AN, Eisler 2016). While these treatment models slightly differ in methods of delivery (e.g. number of treatment phases, focus on engagement, use of formulation etc.) both models align with similar underpinning principles (Baudinet, Simic, and Eisler 2021a; Reinecke and Le Grange 2022). This includes not focussing on the cause of the illness, instead in early treatment there is a strong emphasis on quick behavioral change around eating and weight gain. The family are seen as a key resource, as experts on the young person, and the clinician is active in the process of empowering them to take a lead in supporting their child to recovery. Externalisation of the illness is seen as crucially important in reducing parental guilt and blame, and there is a pragmatic approach to treatment, focussing initially on symptom reduction. Treatment is manualised (e.g. Lock and Le Grange 2012) to support standardisation and dissemination of this evidence‐based approach. Treatment response (defined as an improvement in weight and eating‐related psychopathology) for manualised FBT is on average 75% and average remission rates are about 38% at the end of treatment, leaving approximately 60% of individuals with eating disorder symptomatology (Lock and Le Grange 2019). Byrne and Fursland (2024) highlighted the need for evidence‐based approaches to continue to develop to align with contemporary understanding of eating disorders and their treatment, including our increased awareness and understanding of autism.

Autistic individuals are twice as likely to experience eating disorders compared to non‐Autistic people (Sedgewick, Leppanen, and Tchanturia 2021), and prevalence of Autistic traits in individuals with AN are between 20% and 40% (Mandy and Tchanturia 2015; Westwood & Tchanturia et al. 2017; Spek et al. 2020; Huke et al. 2013). Two recent systematic reviews have reported that Autistic people report poorer experiences of eating disorder treatment, may be at increased risk of inpatient admission and more extensive use of high intensity treatment (Nimbley et al. 2024; Li et al. 2022). Parents and carers have reported frustration at the lack of adaptions to treatment for Autistic people with an eating disorder (Adamson et al. 2020) and clinicians have described a reduced confidence in delivering eating disorder treatments to Autistic individuals (Kinnaird, Norton, and Tchanturia 2017).

In the limited research that has taken place on outcomes of FT‐ED with Autistic young people, this pattern appears to be replicated. Stewart and colleagues (2017) presented outcomes of a specialist child and adolescent eating disorder service delivering FT‐AN as first line intervention and reported comparable physical outcomes and length of treatment across levels of Autistic traits, but a significantly greater use of day patient and inpatient for those with high Autistic traits. Similarly in a case series of FBT, significantly more Autistic young people received intensified care (day programme or inpatient), but there was no significant difference between groups in weight normalisation or successful ending of treatment (Bentz, Pedersen, and Moslet 2022). Furthermore, one of the most replicated markers of poor treatment response is the presence of obsessive‐compulsive and perseverative thinking (Le Grange et al. 2012; Lock et al. 2006; Madden et al. 2015), characteristics which may be present in Autistic populations.

Encouragingly, some adaptations to eating disorder treatment for Autistic individuals are already being considered and implemented. Tchanturia and colleagues (2020) reported reduced length of inpatient admissions following implementation of a clinical care pathway modified for Autistic individuals with AN (PEACE pathway: Tchanturia et al. 2020). Loomes and Bryant‐Waugh (2021) proposed some ways of accommodating FT‐ED for Autistic young people including improving predictability of sessions, consideration of sensory preferences, and preference for routine and sameness in the refeeding process. There is a need to build on this work and begin to explore clinicians' experiences of FT‐ED with Autistic young people, further guiding useful adaptations to the models. The aim of this study is to explore the experiences of clinicians in the delivery of FBT or FT‐AN for Autistic young people with AN, how this differs from delivery to neurotypical young people, and any adaptations currently being implemented.

## 2 Methods

The study received approval from the University of Edinburgh (CAHSS2401/04) and participants gave written informed consent.

### 2.1 Participants

Participants met inclusion criteria if they were healthcare professionals formally trained in eating disorder focused family therapy (specifically FBT or FT‐AN) who had delivered the model to at least one Autistic young person and their family in the past three years. Participants were required to speak English.

### 2.2 Procedures

Participants were recruited through social media, word of mouth, and relevant professional networks. Interested participants contacted the principal investigator via email or completed an online survey included in study adverts. Online interviews were organised via email and a consent form, brief demographics survey and clinical background questionnaire were completed via an online survey. The interviews, facilitated by FD and EC, took place on Microsoft Teams and were recorded with participants' consent. The interviews were guided by a series of questions (see Supporting Information S1: Table S1) considering participants experience of delivery FBT or FT‐AN with Autistic young people, including consideration of underlying principles, specific manualised techniques, and any adaptions to the model. The interview lasted between 43 and 70 min (on average 54 min).

### 2.3 Participant Characteristics

The final sample (*n* = 11) consisted of individuals in a range of clinical roles including Family Therapy (*n* = 2), Nursing (*n* = 5) and Clinical Psychology (*n* = 4) (female *n* = 9, 81%; Mean age 39.5 years, SD: 8.3, range 29–58 years; White British *n* = 9, 81%). Seven participants were based in NHS Scotland, three in NHS England and one from Australia. Participants were trained in FBT (*n* = 8) or FT‐AN (*n* = 3), five had received the basic training, two were accredited practitioners, two accredited supervisors and one a trainer. Years of delivering FT‐AN or FBT ranged between 9 months and 18 years. Participants reported that Autistic, or suspected Autistic, young people ranged from 15% to over half of the total FBT/FT‐AN cases they had worked with. For reasons of confidentiality, demographic information has not been linked to individual participant narratives.

### 2.4 Analysis

The transcripts were analysed using Reflexive Thematic Analysis as described by Braun and Clarke (2021). The researchers took an inductive critical realist approach, being led by the data whilst holding in mind individual perspective of reality (Willig 2022). Microsoft Teams provided verbatim transcriptions which were then checked and anonymised. The six stages of thematic analysis described by Braun and Clarke (2021) were applied to the data. This involved firstly familiarisation with the data where transcripts were read multiple times to allow immersion by FD, IP and EC who then generated initial codes. The researchers organised the codes into initial themes separately, which were then discussed together. Finally, they reviewed each of the themes and subthemes with the larger research group consisting of FBT and FT‐AN trained clinicians, and preliminary results were sent to all participants to confirm accurate representation of their perspective. The final analysis is presented in the results section.

### 2.5 Reflexive Statement

Reflexive Thematic Analysis emphasises the significance of the interpretation and influence of the researcher. FD and EC are clinician researchers experienced in delivering and supervising FBT. They were able to bring their clinical experience to the interviews and analysis. Both researchers value FBT clinically but have also experienced families where this approach has not been successful, including families of some Autistic young people. They were joined in analysis by IP an assistant psychologist who works with individuals with EDs clinically and in research. A peer researcher with lived experience (EM) consulted on the analysis alongside two clinicians trained in FT‐AN (RL) and FBT (LT).

## 3 Results

The Reflexive Thematic Analysis resulted in four themes: (1) Systemic context, (2) Raising potential autism, (3) Autism eating disorder crossover, and (4) Manual versus adaptations (see Table 1). They are discussed with quotes from the transcripts.

**TABLE 1:** Overview of themes.

Theme	Subthemes
1. Systemic context	Neurodivergent parents and carers
	Multiple layers
	Parenting an autistic child
2. Raising potential autism	
3. Autism eating disorder crossover	Differentiating between autism and eating disorders
	Agnostic stance in context
	Autistic empowerment
4. Manual versus adaptations	Tension between evidence‐based practice and potential harm
	Autistic specific considerations

### 3.1 Systemic Context

This theme highlights systemic considerations beyond the young person as an individual, inclusive of three sub‐themes highlighting *Neurodivergent parents and carers, Multiple Layers* of communication that take place within the family and therapeutic relationship, and the prior experience people bring of *Parenting an Autistic child*.

#### 3.1.1 Neurodivergent parents and Carers

When considering FT‐ED for Autistic young people, most clinicians highlighted the need to consider neurodivergence within the family, not just the Autistic young person. Clinicians reflected that some principles of FT‐ED were well aligned with Autistic family's needs.

I'm looking at one person in particular here who's whose parents I think are also … Autistic. And actually, that pragmatic approach, what had worked has worked really well for that family because it was just a case of this is kind of what you need to do here are the reasons you need to do it.

(HCP 11)

However, some of the techniques were noted to be more problematic for Autistic families such as the use of externalizing and circular questions.

When I've been doing FBT with young people who already have a definite diagnosis of autism, you're usually seeing neurodivergence likely in the parents as well… typically has not been diagnosed, so I've found within the FBT model circular questioning can be quite difficult and usually a lot of, and again, this isn't really the young person, this is kind of the parents, who are really always looking for that prescriptive like what do we do? or you know it's kind of like when you're using the principles to guide the family to find solutions and empower them that they actually find that quite stressful….

(HCP1)

This parental preference for instruction over guided discovery led to some clinicians to adapt their communication or stance with families.

I find myself taking more authoritarian stance are ones where I'm wondering whether the child might be Autistic, you know, and also then maybe where one or both of the parents are.

(HCP 9)

This clinician is beginning to reflect on the interaction in communication that takes place between clinicians, young person and parents.

#### 3.1.2 Multiple Layers

Clinicians were able to reflect on different neurotypes within the family and therapeutic relationship and the potential for miscommunication between the young person, family and clinician. Unintentionally, the differences in understanding could lead to families experiencing FT‐ED as punitive.

Where the autism isn't recognised or there's multiple layers of kind of the double empathy problem in action. That can make FBT hard because I think there has to be a good relational base and a shared understanding and a way that it's done so that everyone feels like this is coming from a caring position, not a misattuned kind of punitive position. Yeah and I've just found it can just more easily fall into that latter category with Autistic young people and their families.

(HCP 2)

This included reflections of a neurodivergent therapist about the impact of communication differences and how this can be interpreted

I think sometimes neurodivergent parents are, you know, nothing to get at neurodivergent parents. I’m neurodivergent, I'm a parent, so I'm not criticising, but I do find sometimes, like the communication, I've really got to think about how I’m communicating how my face looks, how I’m coming across. Because, you know, I've never telling them what to do or saying, you know. But it's it can be quite tricky and sometimes they perceive that I'm being negative or giving them into trouble.

(HCP1)

Both clinicians highlighted that misattunement across the therapeutic relationship can lead to perception, or even inadvertent adoption, of a more negative or punitive position for the FT‐ED therapist.

#### 3.1.3 Parenting an Autistic Child

Clinicians reflected on the historical experience of parenting an Autistic young person that families bring to appointments, particularly those with experience of a young person with a demand avoidance profile, and the FT‐ED requirement of a different style of parenting including rapidly engaging in the distressing process of eating.

Sometimes parents have found that stance harder, and if there's if there's such like explicit distress from the young person, that then being able to kind of hold that line of kind of we've made this decision and this is what's going to happen, this is for your best interest I think I found parents can find that harder when they have the Autistic young person.

(HCP5)

Parents do find it tougher and if they already have additional support needs prior to the eating disorder, you know the parents will say that they're there to protect that child.

(HCP1)

Some clinicians reflected on their own experiences and biases and the challenge of continuously holding in mind the experience of the Autistic young person and their family.

It makes me question who I am in the room and that is that kind of I'm not Autistic, I don't have direct experience of Autistic people in my life, so for me, coming into that room and my thinking around parenting, thinking about boundaries and behaviours, and like so I am challenged a lot around that, things that I would just not tolerate.

(HCP3)

A common consideration for Autistic young people and their families was for clinicians to use a separated model of FT‐ED, partly to support open discussions around parenting experiences, but also if a young person was getting overwhelmed within family appointments.

### 3.2 Raising Potential Autism

Clinicians noted most young people present to ED services without autism having been explored before. Contemplating an autism assessment at a point of heighted distress owing to the recent onset of the ED was perceived to be overwhelming for families and clinicians.

We're making the diagnosis or we're suggesting they pursue a diagnosis. And so these families and young people are getting their heads around what it means to be Autistic as well as getting their heads around what it means to have an eating disorder.

(HCP2)

Clinicians describe the tension between exploring whether an individual is Autistic, and potential ethical considerations and negative implications if you do not, when you are still trying to engage the family alongside the urgency of the refeeding process. This was exacerbated by ongoing stigma surrounding autism.

Family did not want to entertain that the potential of autism actually and certainly even just thinking about a young person that probably was Autistic, but family didn't want that diagnosis or assessment.

(HCP3)

One potential solution was the universal screening for autism within eating disorder clinics supporting early discussions and adaptions to treatment in a more transparent manner.

There are some things we're trying to do a little bit more systematically, like the screening, like the reasonable accommodations from the outset, rather than waiting until a patient brings it up.

(HCP2)

### 3.3 Autism Eating Disorder Crossover

#### 3.3.1 Differentiating Between Autism and Eating Disorders

Clinicians highlighted the dilemma of considering autism when a young person is acutely starved and the secondary impacts of this. Clinicians reflected on the struggle to confidently differentiate AN from Autistic traits. They were conscious of the impact of inaccurately assuming certain behaviours could be attributed to the eating disorder or were features of their Autistic experience. This appeared more challenging due to the appreciation that there was a need to respond differently to Autistic traits and disordered eating.

It’s sometimes really hard to differentiate whether that experience is anorexic, the food restriction or the eating, or it comes from neuro, you know, diversity traits and elements where people sometimes are, you know, obsessive about things, obsessive about change. And they have the sensory needs that, you know, that they cannot really eat certain foods. And it's really hard sometimes differentiate what anorexia is and what ASD is in the assessment stage and you don't know how to respond to that.

(HCP 8)

I think working out what's being driven by the eating disorder and what's being driven by their Autistic identity and then of course, there's going to be a crossover. In my experience, that crossover is where the conflict and stuckness kind of happens because it's hard to know whether to embrace that aspect of the young person's kind of character and interactions, or whether it's something that needs to be challenged because it's just so entwined.

(HCP 2)

However, the process of questioning and uncertainty comes at a time when a therapist needs to be clear and containing for a family.

I think sometimes always that question of is this autism or is this anorexia can knock a parent’s kind of confidence and clinicians' confidence.

(HCP5)

#### 3.3.2 Agnostic Stance in Context

Clinicians valued the agnostic stance of FT‐ED, not focussing on the cause of the illness and instead focussing on behavioral change and weight gain, especially when a young person was acutely unwell. However, they felt there was a risk of being blinkered to the impact of Autistic traits on eating disorder presentations.

She was more in tune with that Autistic picture than I was, and it wasn't that I didn't… I suppose for me it was about she needs to eat. So I think that in some ways that agnostic bit maybe does get in the way of actually being mindful of what you're seeing.

(HCP3)

They stressed that there was a need to hold the agnostic principle in relation to the cause of the eating disorder, but a need to differentiate and understand Autistic preferences and traits, formulating how they might interact with the AN, to support adaptations to treatment and promote recovery.

We could spend more time in the beginning understanding the young person as a person, their likes, dislikes, preferences, natural kind of ways of being…. rather than imposing quite a rigid kind of schedule of things on to them. So I guess having a bit more time to formulate in the beginning, with the hope that I suppose that will lead to more meaningful, sustained behaviour change.

(HCP 6)

The value of engaging in this work was noted even when there was an urgency to the young person's care.

I do think just yeah, providing the space even early on in treatment when there is, you know, the priority of life and death issues to still create space, of understanding what's autism and what's the eating disorder.

(HCP 2)

#### 3.3.3 Autistic Empowerment

Clinicians highlighted the need for Autistic young people to have a more active and collaborative role in therapy and to share their experience of being Autistic with the family and clinician.

Even though still trying to be guided by the family and their knowledge of the child, might use the young person to upskill the parents on their Autistic identity and what they need of the parents from that point of view, especially if the diagnosis is new.

(HCP 2)

Equally, clinicians reflected on the need to extend the non‐authoritarian and empowering FT‐ED stance to draw on the expertise that parents bring about their Autistic child.

They've found ways to parent their child with who's Autistic and actually we will be really wanting to draw on those strengths and that knowledge from that family, and even more so to be helpful in thinking about how we get eating disorder recovery in the context of having Autistic child.

(HCP5)

### 3.4 Manual versus Adaptations

Clinicians described being caught between following the manual and developing adaptations for Autistic young people. They felt a *Tension between evidence practice and potential harm*, where they were concerned that deviating or rigidly following a manualised approach may both potentially cause harm. Clinicians who did adjust treatment described their *Autistic specific considerations*.

#### 3.4.1 Tension Between Evidence Practice and Potential Harm

Several clinicians perceived FT‐ED as an adaptable treatment, whereas others inferred a pressure to follow the manual in detail to align with an evidence‐based approach.

I certainly have found that the model in general can be applied well, as long as you are willing to kind of, adapt it I suppose as you go and be considerate, be considerate to whatever the young person in the family, where they're at what they need.

(HCP 11)

Sometimes I've been surprised where, like, I actually have started quite on model and it has just worked for an Autistic young person and family, and actually if I had gone too adapted from the very beginning, perhaps I would have lost the principles of the model or lost kind of actually, the evidence base of that model.

(HCP5)

The tension of between a manualised approach and making adaptions appeared to be related to the potential to cause harm.

We also don't want to cause harm and like I want to help this family recover and, you know, be rid of the illness but actually I also don't want to add even more harm at this stage.

(HCP5)

There were concerns that dogmatically adhering to manualised protocols can lead to harm.

But actually there are there are some situations where it feels like is it, is that always the most help to be incredibly dogmatic and rigid and push, you know, the eating and actually is there something in this distress that we're not attending to and that actually feels quite harmful and horrible for the young person?

(HCP 6)

However, clinicians also expressed fears about adapting therapy to the point they could lose the basic principles of eating disorder treatment, with the potential harm of disrupting recovery via overly accommodating eating disorder behaviors.

It's a kind of slightly paranoid voice in the back of my shoulder of, if I am going to make any adaptations, am I actually appeasing or accommodating this eating disorder versus am I actually accommodating a young person in a helpful way because of their other needs.

(HCP 6)

I think some of it is trying to separate, what's… I'm always suspicious of is this anorexia is excuse and that's where I think that I become quite concerned as a service if we just go with the absolute well, let's adapt everything to do everybody's needs and you know, like ohh, like then you've lost any foundation of what we're trying to do.

(HCP3)

These tensions appeared to be perpetuated by a clinical focus on the need for evidence‐based practice, and clinicians being unable to predict which families FT‐ED may be effective with, leaving them without clear indicators of who may benefit from adaptions.

I think I've really tend to found it nearly like a 50/50 split as to whether it feels like it's a model that suits that young person’s presentation and family, or whether actually it really feels like it's perhaps jarring even with slight adaptations.

(HCP5)

#### 3.4.2 Autistic Specific Considerations

Clinicians identified that a solution to this tension was to make adaptions to meet the needs of Autistic young people and their families, but to adhere to the underlying principles of FT‐ED. In this quote the clinician is highlighting this within the context of externalizing techniques which may be problematic for some Autistic young people and their families.

So generally I would ditch the kind of language externalisation and then just focus on so was such and such like this before eating was a problem and just use kind of time comparisons. To bring into the picture that this particular situation is a result of the eating disorder, not because the young person's chosen there. So I will try to find, yeah, other ways to reduce parental criticism and just totally kind of abandoned the language but hold the philosophy. Yeah. Obviously that the young person's not to blame and to find ways to help the family see that as well. It doesn't just rely on the language aspect.

(HCP2)

It was notable that some clinicians stated that they did not make any adaptions to FT‐ED for Autistic young people, but this appeared to be from a position of believing in person centered adaptions for all young people based on their needs and presentation. Other clinicians noted autism specific adaptions commonly found across a range of psychotherapies, including communication and sensory adaptions and associated tools, for example communication passports and environmental adaptations to reduce sensory overload. However, in general clinicians highlighted that they felt Autistic young people needed more time, both to support communication and information processing within appointments, but also to expect a slower pace of change to reduce overwhelm.

Recognising that we might need to do kind of multiple sessions of appointments rather than trying to do that multi layered assessment in one go.

(HCP2)

The same pace for those very urgent things. But then the pace might be slowed down, but the less urgent things and the goal posts would change as well.

(HCP2)

It was also felt that Autistic young people need a more collaborative approach to reduce the overwhelm associated with making rapid changes.

I think I tried to be more flexible and to give them, and I don't know if this is kind of rightly or wrongly, but try to give them more of a voice in the decision making.

(HCP6)

We need to find ways to still hold the non‐negotiables but be presenting in choice and kind of collaborative tone… I feel like people can get really stuck in saying that this is how it has to be and not feeling like they have permission to slightly change the semantics of how things are set up or even the way that it's set up.

(HCP2)

## 4 Discussion

This paper is the first exploration of clinician's experience of delivering FT‐ED to Autistic young people and their families. The systemic context of this work was apparent with clinicians highlighting the increased potential for neurodivergence within families, the historical experience of parenting an Autistic child, and for therapeutic interactions to be taking place across neurotypes. This pointed to two pressing areas of consideration; firstly, the double empathy problem (Milton 2012), a breakdown in reciprocity and mutual understanding between people with different ways of experiencing the world (e.g., different neurotypes). Within this study, there were concerns from clinicians that this misattunement may result in clinicians adopting an authoritarian therapeutic style, moving away from fundamental principles of FT‐ED (Baudinet, Simic, and Eisler 2021a; Reinecke and Le Grange 2022) and potentially triggering a negative and even punitive cycle of communication with families. This is an important consideration when young people who receive FT‐AN have reported that positive therapeutic relationships and establishing trust, strongly influenced the recovery process (Baudinet et al. 2024). The second significant consideration was that some FT‐ED manualised techniques, for example language of externalization and the use of circular questions, may be at odds with Autistic characteristics, including considerations of cognitive flexibility, ability to manage abstract concepts and perspective taking (Pantazakos 2023). This could also have implications for perpetuating misattunement between therapist, young person and family. This highlights the need to explore whether underpinning mechanisms or techniques may differ across neurotypes, for example we are aware that Autistic families may need a more directed approach with clear communication, scaffolding and guidance, and may struggle with a more open guided self‐discovery approach.

A further area of consideration was the late identification of autism in young people presenting to eating disorder services. There is a diagnostic bias against Autistic girls, where they are more likely to be missed, overlooked or diagnosed late (Loomes, Hull, and Mandy 2017), perhaps due to different behavioural presentations (e.g., Antezana et al. 2019) and/or using compensatory behaviours to mask their social differences (Mandy and Tchanturia 2015). Late diagnosed children are more likely to have high levels of mental health difficulties prior to their autism diagnosis and develop even more severe problems as they enter adolescence (Mandy et al. 2022). There is a higher prevalence of eating disorders in females (van Eeden van Hoeken, and Hoek 2021) and the implications of late identification of autism on the development of disordered eating is worthy of further research. Furthermore, while clinicians were quick to highlight potential stigma in families, further research could also explore the potential of a medicalised or deficit approach to autism, common in healthcare services, and the impact this may have on clinicians' perceptions of autism and concerns about openly discussing early in treatment.

This adds complexity within initial eating disorder assessments where there is a need to differentiate between Autistic traits and an eating disorder. While some researchers have argued that the high prevalence of Autistic traits in eating disorder populations are a behavioural consequence of having an eating disorder (e.g., Treasure 2013) more recent evidence suggest that it more likely represents genuine co‐occurring presentations or risk factor for developing an eating disorder (Adams et al. 2024). Clinicians stress that there is a need to spend more time in FT‐ED understanding Autistic preferences and traits and formulating how they might interact with the eating disorder, to consider appropriate adaptations to treatment. Such approaches should acknowledge the heterogeneity and intersectionality of Autistic experiences and work collaboratively with each Autistic individual when looking to untangle what is their Autistic traits and what is their eating disorder and to provide person centred care.

Finally, a major consideration was the tension clinicians experienced on whether to adhere to a manualised approach or to make adaptions to treatment, underpinned by a fear of causing harm. These reflections mirror past qualitative work with FBT clinicians (Aradas et al. 2019) where practitioners inferred that strict fidelity to a manual was required for evidence‐based practice, and this appeared to have a negative impact on their confidence to tailor interventions to meet a young person's needs. In our study, this tension was navigated by making adaptions which allowed clinicians to adopt a neurodiversity affirming approach to treating the eating disorder, while adhering to the underlying principles of FT‐ED. Such capacity to adapt treatment has previously been identified as a requirement of FT‐ED to *“meet the complex needs that one meets in everyday practice*” (Baudinet, Simic, and Eisler 2021a, 362).

There are limitations associated with this study. The sample size, while appropriate for Reflexive Thematic Analysis, it is quite small and mainly consists of white females. The sample consisted of individuals trained in both FBT or FT‐AN therefore the subtle difference between these models is difficult to capture. For example, FT‐AN places greater emphasis on collaborative formulation (Baudinet, Simic, and Eisler 2021b) than FBT and the impact this has on clinicians' capacity to differentiate autism and eating disorders; make appropriate adaptions to treatment and any associated differences to clinical outcomes would be interesting to explore. Furthermore, the self‐selecting nature of participants means that clinicians with more interest and knowledge of autism may have been more attracted to participate, meaning this self‐selected sample may not be representative of all clinicians delivering FT‐ED.

This study is designed to be the first in a series of studies, with future research exploring Autistic young people and their parents experience of FT‐ED, with the aim of merging this learning to co‐produce appropriate adaptions for the Autistic population. Future research will be required to explore whether these adaptations will improve outcomes for Autistic young people with eating disorders.

In conclusion, this study attempted to go beyond structural or environmental changes to therapy to consider clinicians experience of the process within FT‐ED with Autistic young people and their families. It highlighted the systemic considerations within the therapeutic relationships, the late identification of autism in young people with AN, the struggle clinicians experience in differentiating autism and eating disorders and the perceived tension between manualised practice and clinician confidence in using their skills to make appropriate adaptions to treatment to meet the needs of Autistic young people. This study is an initial step to consider adaptions to the FT‐ED model, with the aim of making eating disorder treatments more effective, accessible and acceptable for Autistic young people and their families.

## Ethics Statement

Ethical approval was obtained from the Research Ethics Committee at the University of Edinburgh (CAHSS2401/04).

## Consent

Informed consent was obtained from all participants.

## Conflicts of Interest

The authors declare no conflicts of interest.

## Supporting information

Table S1

Supporting Information S1

## Data Availability

Data from participant interviews is not publicly available to protect anonymity.
