# Supplementary material for: Clinicians' Experiences of Eating Disorder Focused Family Therapy With Autistic Young People
Source: Eur Eat Disord Rev. 2025 Jan 20;33(4):637–46. doi: 10.1002/erv.3173 (PMC12171668; doi:10.1002/erv.3173)
Supplement: Supplementary file 1 — Table S1 [file ERV-33-637-s001.docx]

| **COREQ (Consolidated criteria for Reporting Qualitative research 32 item checklist** | | | |
| --- | --- | --- | --- |
| **Topic** | **Item** | **Guide questions/Description** | **Page No.** |
| **Domain 1: Research team and reflexibility** |  |  |  |
| *Personal characteristics* |  |  |  |
| Interviewer/facilitator | 1 | Which author/s conducted the interview or focus group? | FD and EC |
| Credentials | 2 | What were the researcher’s credentials?  (E.g., PhD, MD) | FD DClin, EC RN, IP PhD candidate, RL DClin, LT DClin, EM peer researcher, EN PhD |
| Occupation | 3 | What was their occupation at the time of the study? | FD Consultant Clinical Psychologist and Senior Lecturer |
| Gender | 4 | Was the researcher male or female? | Female |
| Experience and training | 5 | What experience or training did the researcher have? | FD Sig ED clinical and research experience, additional qualitative training experience |
| *Relationship with participants* |  |  |  |
| Relationship established | 6 | Was a relationship established prior to study commencement? | FD and EC knew of some participants via professional networks as clinicans |
| Participant knowledge of the interviewer | 7 | What did the participants know about the researcher?  e.g. personal goals, reasons for doing the research | Aware of nature of study and goals of robust exploration of experience |
| Interviewer characteristics | 8 | What characteristics were reported about the inter viewer/facilitator?  e.g. Bias, assumptions, reasons and interests in the research topic | Reasons and interest in research topic |
| **Domain 2: Study design** |  |  |  |
| *Theoretical framework* |  |  |  |
| Methodological orientation and theory | 9 | What methodological orientation was stated to underpin the study? e.g. grounded theory, discourse analysis, ethnography, phenomenology, content analysis | Reflexive Thematic Analysis |
| *Participant selection* |  |  |  |
| Sampling | 10 | How were participants selected? e.g. purposive, convenience,  consecutive, snowball | Purposive |
| Method of approach | 11 | How were participants approached? e.g. face-to-face, telephone, mail,  email | Social media and professional networks |
| Sample size | 12 | How many participants were in the study? | 11 |
| Non-participation | 13 | How many participants were in the study? | 0 |
| *Setting* |  |  |  |
| Setting of data collection | 14 | Where was the data collected? e.g. home, clinic, workplace | Microsoft TEAMS |
| Presence of non-participants | 15 | Was anyone else present besides the participants and researchers? | No |
| Description of sample | 16 | What are the important characteristics of the sample?  e.g. demographic data, date | female n=9, 81%; Mean age 39.5 years, SD: 8.3, range 29–58 years; White British n=9, 81% |
| *Data collection* |  |  |  |
| Interview guide | 17 | Were questions, prompts, guides provided by the authors? Was it pilot  tested? | Yes, pilot took place |
| Audio/visual recording | 18 | Were repeat inter views carried out? If yes, how many? | No |
| Repeat interviews | 19 | Did the research use audio or visual recording to collect the data? | TEAMS video but only audio analysed |
| Field notes | 20 | Were field notes made during and/or after the interview or focus group? | Yes |
| Duration | 21 | What was the duration of the inter views or focus group? | 43 and 70 minutes (on average 54 minutes) |
| Data saturation | 22 | Was data saturation discussed? | Y |
| Transcripts returned | 23 | Were transcripts returned to participants for comment and/or correction? | N |
| **Domain 3: Analysis and findings** |  |  |  |
| *Data analysis* |  |  |  |
| Number of data coders | 24 | How many data coders coded the data? | 2 and 3^rd^ consultant |
| Description of the coding tree | 25 | Did authors provide a description of the coding tree? | Y |
| Derivation of themes | 26 | Were themes identified in advance or derived from the data? | Derived from data |
| Software | 27 | What software, if applicable, was used to manage the data? | N-Vivo |
| Participant checking | 28 | Did participants provide feedback on the findings? | No |
| *Reporting* |  |  |  |
| Quotations presented | 29 | Were participant quotations presented to illustrate the themes/findings?  Was each quotation identified? e.g. participant number | Y |
| Data and findings consistent | 30 | Was there consistency between the data presented and the findings? | Y |
| Clarity of major themes | 31 | Were major themes clearly presented in the findings? | Y |
| Clarity of minor themes | 32 | Is there a description of diverse cases or discussion of minor themes? | Y |

Developed from: Tong A, Sainsbury P, Craig J. Consolidated criteria for reporting qualitative research (COREQ): a 32-item checklist or interviews and focus groups. International Journal for Quality in Health Care. 2007. Volume 19, Number 6: pp. 349 – 357
