# Supplementary material for: Clinicians' Experiences of Eating Disorder Focused Family Therapy With Autistic Young People
Source: Eur Eat Disord Rev. 2025 Jan 20;33(4):637–46. doi: 10.1002/erv.3173 (PMC12171668; doi:10.1002/erv.3173)
Supplement: Supplementary file 2 — Supporting Information S1 [file ERV-33-637-s002.docx]

**Supplemental Infomration 1**

**Clinicians interview schedule**

*Experiences of FT-ED with Autistic young people*

Thank you for agreeing to take part in this interview. We are interested in experiences of eating disorder focused family therapy with Autistic young people and their families. There is a lack of research on the experience of traditional treatment of eating disorders with Autistic young people. To develop a better understanding, we are seeking to gather experiences of delivering or receiving this treatment for eating disorders with Autistic young people and their families. We will ask you about your clinical experience and perspective on delivering FBT or FT-AN to this population.

We expect the interview to last between 30 minutes and an hour, but we can stop to take a break at any point, and you can finish the interview at any time without giving an explanation.

[If participant has consented to be recorded, switch on the recorder]

May I double-check that you are happy for this interview to be recorded?

[Continue recording, if participant has confirmed their consent.]

**Interview questions**

🡪 To begin with, would you be able to tell me about your experience working in mental health services for young people?

***Prompts***

- What is your role?
- How long have you been working in the service?
- Would you be able to tell me about your experience with young people? And young people with eating disorders?
- How long have you been using FBT or FT-AN and how do you find using this treatment model?

🡪 You have explained that you are trained in FBT/FT-AN and have delivered it for eating disorders with Autistic young people and their families. During this interview I ask about your experience of delivering FBT/FT-AN, and the use of principles and techniques with Autistic young people with Anorexia Nervosa and Bulimia Nervosa.

In general, how have you found using FBT/FT-AN with Autistic young people (or young people you suspect may be Autistic) and their families?

***Prompts***

- What did you feel to be to be useful for Autistic young people and their families?
- What did you feel to be unhelpful for Autistic young people and their families?

🡪 Prior to starting there is typically an initial eating disorder assessment. Would you be able to tell me about your experience of this assessment process with Autistic young people with eating disorders, that are being considered for FBT/FT-AN?

***Prompts***

- - How does this differ from neurotypical young people with eating disorders?
- Is there any extra complications or considerations in the assessment of Autistic young people and their families? FU – what do you ask further information on? What do you change or adapt from a routine eating disorder assessment?
- How do you manage young people without a diagnosis of autism but who you suspect may be Autistic? FU – how and when do you decide to progress with an assessment of autism? Do you use any screening tools?
- Do the first line psychoeducation or resources that you offer differ if you are assessing an Autistic young person? FU- if you offer different resources, what do you offer and why?
- Are there any considerations you would account for when deciding whether to offer FBT/FT-AN to an Autistic young person and their family? What would you offer if you think FBT/FT-AN is not suitable?

🡪 There are several assumptions or principles that underpin FBT/FT-AN, and we are interested in how you experience these in the context of treating Autistic young people with eating disorders. One of these assumptions is that the therapist should hold an agnostic view of the cause of the illness where they do not focus on exploring causes of the eating disorder.

How did you experience this as a clinician working with an Autistic young person and their family?

***Prompts***

- Can you think of any challenges or helpful parts of this stance that were experienced by yourself, the Autistic young person, or their family regarding this aspect of FBT/FT-AN?
- Does the implementation of this differ than if it was a neurotypical young person?
- Did you make any adaptations or changes to this aspect of FBT/FT-AN due to the knowledge that the young person is, or may be, Autistic?

🡪 Another principle that underpins FBT/FT-AN, is the non-authoritarian therapeutic stance. The therapist is seen as an expert on eating disorders and on treatment, and the parents are seen as the expert on their child and their family. The therapist is active in treatment, providing psychoeducation and guidance, but generally does not tell what the parent to do or exactly how to feed their child.

How did you experience this as a clinician working with an Autistic young person and their family?

***Prompts***

- Can you think of any challenges or helpful parts of this stance that were experienced by yourself, the Autistic young person or their family regarding this aspect of FBT/FT-AN?
- Does the implementation of this differ than if it was a neurotypical young person?
- Did you make any adaptations or changes to this aspect of FBT due to the knowledge that the young person is, or may be, Autistic

🡪 A third principle that underpins FBT/FT-AN, is parental empowerment, building parents confidence in their role as the primary agent for change in the recovery process. This would include the confidence to accomplish the task of refeeding, to stand firm in the event of distress with this and to not second guess themselves.

How did you experience this as a clinician working with an Autistic young person and their family?

***Prompts***

- Can you think of any challenges or helpful parts of this stance that were experienced by yourself, the Autistic young person, or their family regarding this aspect of FBT/FT-AN?
- Does the implementation of this differ than if it was a neurotypical young person?
- Did you make any adaptations or changes to this aspect of FBT/FT-AN due to the knowledge that the young person is, or may be, Autistic?

🡪 A further principle in FBT/FT-AN is externalisation, separating the illness and the adolescent. How did you experience this as a clinician working with an Autistic young person and their family?

***Prompts***

- Can you think of any challenges or helpful parts of this stance that were experienced by yourself, the Autistic young person, or their family regarding this aspect of FBT/FT-AN?
- Does the implementation of this differ than if it was a neurotypical young person?
- Did you make any adaptations or changes to this aspect of FBT/FT-AN due to the knowledge that the young person is, or may be, Autistic?

🡪 FBT/FT-AN takes a pragmatic approach, focusing in on eating disorder symptom reduction (e.g. weight restoration) and for this to take place rapidly in initial phases of FBT/FT-AN. How did you experience this as a clinician working with an Autistic young person and their family?

***Prompts***

- Can you think of any challenges or helpful parts of this stance that were experienced by yourself, the Autistic young person, or their family regarding this aspect of FBT/FT-AN?
- During the first phase of FBT the focus is on a return to eating patterns that were premorbid the eating disorder and/or typical of the family, how did you or the family experience this?
- Does the implementation of this differ than if it was a neurotypical young person?
- Did you make any adaptations or changes to this aspect of FBT/FT-AN due to the knowledge that the young person is, or may be, Autistic?

There are several key features of FBT/FT-AN and we are keen to understand how you experienced these elements with an Autistic young person and their family

- How did you experience introducing weekly weighing as part of FBT/FT-AN, plotting this on weight chart and sharing this openly with the family, and how was this accepted?

***Prompts***

- Did you make any adaptions or changes for this aspect of FBT/FT-AN?
- Another key feature of phase 1 of FBT is the family meal, how did you find the facilitating the family meal with Autistic young people and their families? *(please note that this question is only relevant to those trained in Lock’s FBT model)*

***Prompts***

- Can you think of any challenges that were experienced by yourself, young person or their family which were particularly experienced during the family meal?
- Did you make any adaptions or changes to this aspect of FBT?

🡪 During middle phase of FBT/FT-AN, clinicians facilitate families handing eating back over to their young person. How was do you experience this with Autistic young people?

***Prompts***

- Can you think of any challenges that were experienced by yourself, young person or their family which were particularly experienced during the middle phase of FBT/FT-AN?
- Did you make any adaptions or changes to this aspect of FBT/FT-AN?

🡪 In the final phase of FBT/FT-AN the focus is on exploring adolescent issues, restoring a family dynamic without the eating disorder and ending FBT/FT-AN. How was facilitating this final phase of FBT/FT-AN and the focus on adolescent issues for Autistic young people?

***Prompts***

- Can you think of any challenges that were experienced by yourself, young person or their family during the final phase of FBT/FT-AN?
- Did you make any adaptions or changes to this aspect of FBT/Ft-AN?
- Based on your clinical experience, what can be done to improve FBT/FT-AN for Autistic young people?

***Prompts***

- Is there anything that you would have liked to be able to offer as an alternative?
- We are coming to the end of the interview; do you have any last comments? (Including any suggestions for services or suggestions for interview questions?)

**Ending**

Thank you for participation in our study, I will now stop the recording.
